# Intelligence

**Published:** 1810-09

**Authors:** 


### INTELLIGENCE.

JACKSONIAN PRIZE.—The Prize-Subject for the year 1811, for the premium of 10l. to the Author of the best Dissertation on a practical subject in Surgery, is "Wounds and Diseases of Arteries and Veins."—Candidates must be Members of the College, not on the Court of Assistants.—The Dissertations must be in English: and the number and importance of Facts will be considered the chief points of excellence in the compositions.—Each Dissertation must be distinguished by a Motto or Device; but no Dissertation, Motto, or Device, must be in the hand writing of the author.—Every Dissertation must be accompanied by a paper, sealed up (not with the seal of the author) containing the Name and Residence of the Author; and having, on the outside, a Motto or Device (not in his own hand-wring), corresponding with the Motto or Device on the Dissertation.—Dissertations must be addressed to the Secretary, and delivered at the College before Christmas Day, 1811.—The adjudications will be in the month of April, 1812. —The Prize-Dissertation will be preserved in the library of the College.—The unapproved Dissertations, with their correspondent unsealed papers, will be returned, upon authenticated applications. Every unapproved Dissertation, which shall remain three years unclaimed, and its correspondent paper, unopened, will be burnt in the presence of the Jacksonian Committee.—The Prize-Subject for the present year, 1810, is "The Bite of a Rabid Animal;" Dissertations upon which must be delivered before Christmas Day next.

---

Mrs. Mary Stokes was lately admitted into St. Thomas's Hospital, in consequence of an injury she had received from a cart-wheel going over her neck; a tumour immediately formed, extending from the angle of the lower jaw to the clavicle on the left side, which increased rapidly. Blood was immediately taken from the arm, and when put in a horizontal posture, suffocation would have been brought on; the pulse was full and strong till she expired, which occurred a few minutes after admission into the Hospital.

Upon examination, a large coagulum of venous blood was found occupying the situation of the left internal jugular vein, and this extensively lacerated. The tranverse process of the 7th cervical vertebra was fractured and detached, also the inferior cornuæ of the thyroid cartilage were fractured, but there was no communication into the trachea. On the right side of the neck was found a fracture of the cervix of the first rib, and considerable laceration of the contiguous parts.

UNIVERSITY OF GLASGOW.

The Medical Lectures in the University of Glasgow, will begin on Tuesday the 6th of November, at the following hours.

Dietetics, Materia Medica, and Pharmacy, by Dr. MILLAR, at ten o'clock in the forenoon.

Midwifery, by Mr. TOWERS, at eleven.

Theory and Practice of Physic, by Dr. FREER, at twelve.

Anatomy and Surgery, by Dr. JEFFRAY, at two o'clock afternoon.

Chemistry and Chemical Pharmacy, by Dr. CLEGHORN, at seven.

Dr. BROWN will commence his Lectures on Botany, about the beginning of May next.

The following Gentlemen have obtained the degree of Doctor in Medicine, from this University, within the last twelve months.

| | |
|---|---|
| Mr. MICHAEL M'TURK, | *from Scotland.* |
| Mr. ALEXANDER MARSHALL, | *from Scotland.* |
| Mr. ALEX. JAMES BUCHANAN, | *from Scotland.* |
| Mr. WILLIAM D. NELSON, | *from Scotland.* |
| Mr. JAMES BALDERSTONE KIRK, | *from Scotland.* |
| Mr. RICHARD KENNEDY, | *from East Indies.* |

---

ST. THOMAS'S and GUY'S HOSPITALS.

The Autumnal Courses of Lectures at these adjoining Hospitals, will begin the 1st of October, as follows.

*At Thomas's.*—Anatomy, and the Operations of Surgery, by Mr. CLINE, and Mr. COOPER. Principles and Practice of Surgery, by Mr. COOPER.

*At Guy's.*—Practice of Medicine, by Dr. BABINGTON and Dr. CURRY. Chemistry, by Dr. BABINGTON, Dr. MARCET, and Mr. ALLEN. Experimental Philosophy, by Mr. ALLEN. Theory of Medicine, and Materia Medica, by Dr. CURRY and Dr. CHOLMELEY. Midwifery, and Diseases of Women and Children, by Dr. HAIGHTON. Physiology, or Laws of the Animal Œconomy, by Dr. HAIGHTON. Structure and Diseases of the Teeth, by Mr. FOX.

N. B. These several Lectures are so arranged, that no two of them interfere in the hours of attendance; and the whole is calculated to form a *Complete Course of Medical and Chirurgical Instruction.* Terms and other Particulars may be learnt at the respective Hospitals.

---

Dr. BUXTON'S Autumnal Course of Lectures on the Practice of Medicine, will be commenced on Monday the 1st of October, at the *London Hospital.*

Mr. A. CARLISLE will begin his Course of Lectures on the Art and Practice of Surgery, on Thursday October 4, 1810, at eight o'clock in the evening, at his house in Soho Square. The Introductory Discourse will be open to all Students.

Mr. CARPUE will commence his Lectures on Anatomy, Surgery, &c. on the 1st of October.—Particulars at Mr. Carpue's, No. 50, Dean Street, Soho.

Dr. CLARKE's and Mr. CLARKE's Lectures on Midwifery, and the Diseases of Women and Children.—The Winter Course of these Lectures will commence on Friday the 5th of October, at the house of Mr. Clarke, No. 10, Upper John Street, Golden Square. The Lectures are read every day from a quarter past ten o'clock in the morning, till a quarter past eleven, for the convenience of Students attending the Hospitals. The Students will have Labours, when properly qualified.—For Particulars, apply to Dr. Clarke, No. 1, New Burlington Street, or to Mr. Clarke, No. 10, Upper John Street, Golden Square.

Dr. CLOUGH, Physician Manmidwife to the St. Mary-le-bone General Dispensary, &c. will on Monday the 8th of October, at ten in the morning, commence his Winter Course of Lectures on the Theory and Practice of Midwifery, including the Diseases of Women and Infants, at his Lecture Room, 68, Berner's Street. The evening Courses as usual, Tuesdays, Thursdays, and Saturdays, at seven, during the Season. For Syllabus and Prospectus of the Course, apply as above.

Dr. CLUTTERBUCK will begin his Autumn Course of Lectures on the Theory and Practice of Physic, Materia Medica, and Chemistry, on Friday the 5th of October, at ten o'clock in the morning precisely, at his house, No. 1, Crescent, New Bridge Street; where further particulars may be had, or at the General Dispensary, Aldersgate Street. The Lectures are given daily; viz, Theory and Practice, Mondays, Wednesdays, and Fridays.—Materia Medica and Chemistry, on Tuesdays, Thursdays, and Saturdays, at the same hour.

Dr. DENNISON and Dr. BYAM DENNISON will commence their Course of Lectures on the Theory and Practice of Midwifery and the Diseases of Women and Children, at the *London Hospital*, on Monday, October 8, at eleven o'clock. For particulars, enquire at Dr. Dennison's, 37, Broad Street Buildings, or of Mr. Price, at the Hospital.

Dr. HOOPER and Dr. AGER will commence their Lectures on the Theory and Practice of Physic, the Materia Medica, and Chemistry, on Monday October the 1st, 1810, at the Theatre in Cork Street, Burlington Gardens, at eight o'clock in the morning. A Prospectus with Particulars may be had, by applying to Dr. Hooper, at his house, No. 21, Saville Row.

Mr. MOOR, Surgeon-dentist to her royal highness the Duchess of York, will commence a Course of Lectures on the Structure and Diseases of the Teeth, on the 11th of October, in which will be explained the complete Practice of the Dentist. Further particulars may be known at his house, No. 6, Palsgrave Place, Temple.

Dr. RAMSBOTHAM will commence his Lectures on the Science and Practice of Midwifery on Wednesday, Oct. 10, at his house, No. 9, Old Jewry, which will be illustrated by anatomical preparations and casts from a celebrated valuable Museum. The Prospectus may be had as above.

Dr. REID's next Course of Lectures, on the Theory and Practice of Medicine, will commence on Monday the 8th of October; and will conclude on Monday the 31st of December. The Lectures will be given at nine o'clock in the morning, on Mondays, Wednesdays, and Fridays, at Dr. Reid's house, Grenville Street, Brunswick Square; where further Particulars may be known.

Dr. JAMES ROBERTON, Member of the Royal College of Physicians, and Physician to the British Lying-in Hospital, will commence his next Course of Lectures on the Principles and Practice of Midwifery, and the Diseases of Women and Children, on Thursday the 4th of October, at a Quarter past ten in the morning, at Mr. Bell's Museum, No. 10, Leicester Street, Leicester Square. —House pupils will have the privilege of attending Mr. Bell's Lectures, and Dr. Roberton's Demonstrations of Anatomy and Dissection. For particulars enquire at Dr. Roberton's house, 14, Old Burlington Street, Burlington Gardens, or at the Museum, Leicester Street, where the Lectures will be delivered.

Mr. SHUTE will commence his Winter Course of Lectures on Anatomy, Physiology, and the Principles and Operations of Surgery, on Monday the 1st of October, at the Anatomical Theatre, Bristol, at eight o'clock in the morning.

Mr. TAUNTON will commence his Autumnal Course of Lectures on Anatomy, Physiology, Pathology, and Surgery, on Saturday, October 6, 1810, at eight o'clock in the evening precisely, which will be continued every Tuesday, Thursday, and Saturday, at the same hour. Particulars may be had by applying to Mr. Taunton, Greville Street, Hatton Garden.

Dr. TUTHILL will begin his Winter Course of Lectures on the Practice of Physic, and on the Laws and Operations of Chemistry, on Monday October the 1st, 1810. Further Particulars may be known by applying to Dr. Tuthill, at his house in Soho Square, where the Lectures are delivered.

Mr. STEVENSON, Great Russell Street, Bloomsbury, Pupil of the late Mr. SAUNDERS, has nearly ready for publication, a practical Work on a very prevalent Disease of the Eye.

